# Iconic gestures prime words: comparison of priming effects when gestures are presented alone and when they are accompanying speech

**DOI:** 10.3389/fpsyg.2013.00779

**Published:** 2013-10-21

**Authors:** Wing-Chee So, Alvan Low Yi-Feng, De-Fu Yap, Eugene Kheng, Ju-Min Melvin Yap

**Affiliations:** ^1^Department of Educational Psychology, The Chinese University of Hong KongHong Kong, China; ^2^Department of Psychology, National University of SingaporeSingapore, Singapore; ^3^Department of Psychology, University of ChicagoChicago, IL, USA

**Keywords:** co-speech gestures, cross-modal priming, lexical decision, language processing

## Abstract

Previous studies have shown that iconic gestures presented in an isolated manner prime visually presented semantically related words. Since gestures and speech are almost always produced together, this study examined whether iconic gestures accompanying speech would prime words and compared the priming effect of iconic gestures with speech to that of iconic gestures presented alone. Adult participants (*N* = 180) were randomly assigned to one of three conditions in a lexical decision task: Gestures-Only (the primes were iconic gestures presented alone); Speech-Only (the primes were auditory tokens conveying the same meaning as the iconic gestures); Gestures-Accompanying-Speech (the primes were the simultaneous coupling of iconic gestures and their corresponding auditory tokens). Our findings revealed significant priming effects in all three conditions. However, the priming effect in the Gestures-Accompanying-Speech condition was comparable to that in the Speech-Only condition and was significantly weaker than that in the Gestures-Only condition, suggesting that the facilitatory effect of iconic gestures accompanying speech may be constrained by the level of language processing required in the lexical decision task where linguistic processing of words forms is more dominant than semantic processing. Hence, the priming effect afforded by the co-speech iconic gestures was weakened.

## Introduction

We gesture when we talk. Among different types of gestures, iconic gestures display physical properties and movements of objects or actions. As suggested by McNeill (1992; p. 155), iconic gestures represent thought or are so-called “*material carriers of thinking.*” Previous research has shown that iconic gestures often convey semantic information relevant to that in speech (e.g., McNeill, 1992; Kita and Özyürek, 2003; Özyürek et al., 2005). For example, a speaker extends his little finger and thumb (with thumb pointing up and little finger pointing down) and puts his hand next to his ear while saying, “I'll call you tonight.” This iconic gesture resembles the action of talking on a phone and reinforces the semantic content expressed in speech.

The question of interest is whether *encoding* an iconic gesture would activate semantically related words. This question can be addressed in a cross-modal semantic priming experiment. Semantic priming refers to an increase in speed or accuracy in responding to a target stimulus when it is preceded by a semantically related stimulus, compared to when it is presented by a semantically unrelated stimulus (McNamara, 2005). For example, in the primed lexical decision task, participants initially view a prime word (e.g., DOG) followed by a target stimulus. This target is either a word, semantically related (e.g., CAT) or unrelated to the prime (e.g., HAT), or a non-word (e.g., NOM). Participants then determine whether the targets are words or non-words. Substantial research has shown that participants respond faster to semantically related words than to semantically unrelated words (see Neely, 1991 and McNamara, 2005 for reviews).

In cross-modal priming experiments, which examine whether there is a semantic link between iconic gestures and lexical representations, word primes in the semantic priming paradigm are replaced by iconic gestures. To date, two studies have explored cross-modal priming with gesture primes. An initial study by Bernardis et al. (2008) reported that iconic gestures did *not* prime semantically related words. They selected 40 video clips of gestures that were paired with 80 word targets, with half of the targets semantically related to the gestures and the rest unrelated. Participants in the gesture group were asked to view the gesture video clips (each lasting ~3600 ms), and then to read the following word targets as rapidly as possible. Participants in the baseline group were also told to name the same word targets but these words were presented in isolation (i.e., without gesture clips). The difference in response time between the two groups was then computed, where response time refers to the time elapsed between the presentation of word targets and the onset of the participant's vocal response. The findings indicated that naming target words that were semantically related to preceding gestures was not significantly faster than naming target words that were presented alone, possibly suggesting that iconic gestures did not prime semantically related words.

However, the findings in Bernardis et al.'s (2008) study should be interpreted with caution for two reasons. First, naming target words presented alone and naming the same words preceded by semantically related gestures might involve different processing demands. Specifically, it was very likely that participants in the gesture group needed additional amount of time to encode target words that were preceded by semantically related gestures because these gestures were informative and thus required time to process. However, this extra time and effort in processing target words was not relevant in the baseline group. Hence, comparing the response time of participants in the baseline group to that of the gesture group might eventually attenuate the facilitatory effects of gestures (see Yap et al., 2011 for more discussion). Indeed, one should calculate the overall priming effect, which is the difference in response time to semantically-related words (e.g., CAT—DOG) and that to semantically-unrelated words (e.g., HAT—DOG) (Neely, 1977; Jonides and Mack, 1984). As such, Yap et al. (2011) re-examined Bernardis et al.'s (2008) results and found a significant overall priming effect. Specifically, participants responded faster when reading words that were semantically related to the preceding gestures than when reading words that were not semantically related to the preceding gestures.

However, there is still an alternative explanation for the significant overall priming effect. The average duration of each gesture clip in their study was longer than 3000 ms, which might in principle provide participants with sufficient time to strategically recode gestures with verbal labels. This pertains to the second limitation in Bernardis et al.'s study. Labeling gestures would in turn activate semantically-related words, which might serve as lexical primes. Henceforth, the overall priming effect in the naming task could be attributed to lexical primes instead of gesture primes. In order to prevent participants from naming gesture primes, one should keep each gesture clip shorter than 3000 ms.

Based on the limitations described above, Yap et al. (2011) conducted another cross-modal priming experiment wherein they administered a lexical decision task. In both experiments in Yap et al.'s study, they presented participants video clips of iconic gestures that carried semantic meaning (e.g., a two-hands flapping gesture) followed by lexical targets which were either words (e.g., “bird,” “brush”) or non-words (“blurds”) and then asked participants to make lexical decisions. The iconic gestures clips lasted for 3500 ms (Experiment 1; also see Bernardis et al., 2008) as well as 1000 ms (Experiment 2). The results in both experiments showed that participants responded faster and more accurately to words which were semantically related to gestures (e.g., “bird,” which is semantically related to a two-hands flapping gesture) than to words which were not semantically related to gestures (e.g., “bad”). These findings suggest that iconic gestures facilitate recognition of semantically related words. They are also consistent with the theories which suggest that our memorial representations are encoded in multiple formats or levels, including gestures and their lexical affiliates (Krauss, 1998) and gesture and language form *single* system within a unified conceptual framework (McNeill, 1992, 2005).

However, a critical issue remains unaddressed in both Bernardis et al. (2008) and Yap et al.'s (2011) studies. These two studies examined gestures when they *were* presented alone rather than co-occurring with speech. However, gestures themselves are idiosyncratic as their forms do not conform to any standard, thereby making it difficult for participants to interpret their meaning, especially when they are presented in an isolated manner (Feyereisen et al., 1988; Krauss et al., 1991, 2000). Therefore, some researchers have concluded that gestures can only be appropriately interpreted together with accompanying speech (Krauss et al., 1991, 2000). Having said that, the iconic gesture primes examined in Yap et al.'s (2011) study were well recognized by majority of participants (at least 70% agreement rate in a separate norming study). Hence, it is possible that some iconic gestures, compared to others, possess a more direct and transparent relationship with their meaning, thereby allowing them to be recognized more easily (Beattie and Shovelton, 2002) even in the absence of speech.

Nevertheless, iconic gestures and speech are almost always produced together in a natural communicative setting. According to McNeill (2005), 90% of gestures are produced in tandem with speech. In addition, both gestural and spoken modalities are tightly integrated temporally, semantically and pragmatically (McNeill, 1992; Kita, 2000; So et al., 2009). Temporally, Morrel-Samuels and Krauss (1992) found that gestures are initiated either prior to or simultaneously with the onset of their lexical affiliate, thus suggesting a tight temporal link between the gesture and language systems during communication. Semantically, So et al. (2009) reported that participants are more likely to identify a referent in gestures when the referent is also lexically specified in speech than when it is not, thus suggesting a parallel semantic relationship between gestures and their co-occurring speech. Pragmatically, Kita (2000) proposed that gestures facilitate speaking by packaging preverbal spatial-motoric information into units suitable for verbalization (the Information Packaging Hypothesis).

The wealth of evidence has demonstrated the tight integration of gesture and speech in the realm of language production. One significant implication of these findings is that people are exposed to gestures and their accompanying speech during language comprehension. As a result, it is necessary to test for priming when gestures are paired with speech, i.e., studying speech and gestures as a combined system rather than as separate modalities.

In the present study, we aimed to explore the interplay between co-speech iconic gestures and the lexical processing system. Specifically, we examined whether iconic gestures accompanying speech would prime semantically related words and whether such priming was comparable in size to that produced by iconic gestures presented alone in a lexical decision task (Yap et al., 2011). We also set out to examine whether the priming effect of iconic gestures accompanying speech was comparable to that of speech alone. The findings in the present study will shed more light on the influence of iconic gestures (either presented alone or co-occurring with speech) on the word recognition process.

We used three types of primes in the present study; iconic gestures alone (Gestures-Only condition), speech alone (Speech-Only condition), and iconic gestures accompanying speech (Gestures-Accompanying-Speech condition). The prime stimuli in the Gestures-Only condition were identical to those in (Yap et al.'s 2011) study. Those in the Speech-Only condition were auditory tokens that labeled iconic gestures examined in the Gestures-Only condition. Those in the Gestures-Accompanying-Speech condition contained the simultaneous coupling of gesture clips in the Gestures-Only and auditory vocal tokens in the Speech-Only condition. Hence, the iconic gestures and auditory vocal tokens were conveying the same meaning. However, it might not be naturalistic for not having a speaker producing gestures and speech in a synchronized manner. It could be a potential limitation in this condition.

In line with (Yap et al.'s 2011) study, we predicted that iconic gesture primes presented alone would result in faster response times to semantically related than to semantically unrelated words. We also predicted that words preceded by semantically related auditory primes presented alone should be responded faster than those preceded by semantically unrelated auditory primes. This expectation is in line with other cross-modal studies in the literature (Holcomb and Neville, 1990; Holcomb and Anderson, 1993). For example, a study by Holcomb and Neville (1990) showed that auditory primes yielded faster response rate to visually presented related words than to unrelated words in both behavioral (response latencies) and electrophysiological (event-related potentials) experiments.

The focus of our interest was the priming effect of iconic gestures accompanying speech, which was not examined in previous research. We predicted that the priming effect of co-speech iconic gestures would be significant because these gestures were the same as those in the Gestures-Only condition. However, would the priming effect in the Gestures-Accompanying-Speech condition be stronger than that in the Gesture-Only condition, or vice versa?

On one hand, processing iconic gestures that are co-occurring with speech would induce a *stronger* priming effect than processing the same iconic gestures on their own and processing speech alone. According to the Integrated Systems Hypothesis (Kelly et al., 2010), iconic gestures and their co-occurring speech are tightly integrated in the process of language comprehension (for a review, see Kelly et al., 2008). This gesture-speech integration in language comprehension is driven by obligatory interaction between speech and its co-occurring iconic gestures. According to this view, participants would automatically process the information presented in speech while taking into account the information presented in gestures (Kelly et al., 2010). This hypothesis has found empirical support in recent studies (e.g., Kelly et al., 2004; Holle and Gunter, 2007; Özyürek et al., 2007; Wu and Coulson, 2007; Hubbard et al., 2009). For example, previous studies which used event related potentials have found that iconic gestures are integrated with speech during the process of language comprehension (Kelly et al., 2004; Özyürek et al., 2007). In the present study, iconic gestures and their co-occurring speech conveyed congruent semantic meaning. Hence, the presence of the accompanying speech would reinforce and consolidate the meaning of iconic gestures (Krauss et al., 2000), thereby yielding a stronger priming effect, when compared to a condition when iconic gestures were presented on their own.

Similarly, iconic gestures accompanying speech should yield stronger priming effect than speech alone. There is abundant behavioral evidence reporting that co-speech gestures aid language comprehension and memory (e.g., Cohen and Otterbein, 1992; Beattie and Shovelton, 1999; Kelly et al., 1999; Feyereisen, 2006; So et al., 2012). Physiological studies have also documented that gestures in general (iconic gestures and speech beats) influence speech perception and modulate neural activities (e.g., Dick et al., 2009; Biau and Soto-Faraco, 2013). Other studies have also shown benefits of gesture and speech integration on comprehension (Kelly et al., 2004; Wu and Coulson, 2005; Özyürek et al., 2007). Although the communicative power of gestures is greater when gestures are non-redundant with co-occurring speech than when they are completely redundant (Hostetter, 2011), presenting redundant iconic gestures would still direct listeners' attention to the accompanying speech (Kelly and Goldsmith, 2004), thereby enhancing the priming effect.

On the other hand, the priming effect of co-speech gestures might be weaker than that of iconic gestures presented on their own. As mentioned earlier, speech helps reinforce and consolidate the meaning of co-occurring iconic gestures gestures (Krauss et al., 2000). As opposed to iconic gestures accompanying speech (and speech alone), those presented alone are far less constrained in their meaning. This is because, as compared to words, iconic gestures might be seen as more idiosyncratic and less associated with conventional labels (McNeill, 1992, 2000). Due to the absence of standardized forms and meanings, iconic gestures presented alone might be semantically related to a wider range of words or concepts, compared to iconic gestures accompanying speech and speech presented alone, henceforth strengthening its priming effect.

In addition, the priming afforded by speech accompanied by iconic gestures might not be necessarily stronger than that afforded by speech presented alone. Much of the previous work supporting the Integrated Systems Hypothesis have examined the gesture-speech integrated relationship at the “semantic” level, which emphasizes relatively high level language processing. In many of these studies, participants were required to answer questions pertaining to their understanding of the gestures and speech (Beattie and Shovelton, 1999; Kelly et al., 1999, 2004; Dick et al., 2009) or to determine whether gestures and their accompanying speech conveyed congruent or incongruent semantic meaning (Kelly et al., 2010, 2012). This leaves open the possibility that iconic gestures, which are accompanying speech, enhance higher-level *semantic* processing, but not lower-level *word form* processing. This argument has found some empirical support. In Kelly and Lee's study (2012), native English-speaking participants were taught the meaning of novel Japanese words, which were accompanied or not accompanied by congruent iconic gestures. They then performed two tasks, a semantic task (where they chose the correct English translation of a Japanese word) and a phonetic task (where they determined whether the presented Japanese word contained a geminate or singleton). Their findings showed that co-speech iconic gestures facilitated learning in the semantic task but not in the phonetic task, suggesting that co-speech iconic gestures might not aid processing of phonetic forms (also see Hirata and Kelly, 2010 for speech beats).

Like the phonetic task, the reliance on semantic processing is relatively low in the lexical decision task. In the lexical decision task, participants are attempting to discriminate real words from orthographically similar distracters. While lexical semantic variables such as concreteness, imageability and valence affect lexical decision times, emphasis is placed more on the forms of words than their meanings when doing this task. According to the Theory of Language and Situated Simulation (LASS; Barsalou et al., 2008), making lexical decisions (i.e., discriminating words from non-words) can be based on processing linguistic forms alone without accessing deep conceptual meaning. This perspective is consistent with findings from Pexman et al.'s (2008) study, which indicated that the lexical decision task focused considerably more on word form than on word meaning, whereas the semantic classification task (i.e., discriminate words from different semantic categories, e.g., animate vs. inanimate) focused more on word meaning than on word form. In short, the findings from Kelly and Lee (2012) and Pexman et al. (2008) suggest that iconic gestures accompanying speech might not facilitate word recognition when making lexical decision.

Related to this line of argument, the priming effect of speech accompanied by iconic gestures might yield a *comparable* priming effect to that of speech presented alone. According to the LASS theory (Barsalou et al., 2008), the processing of linguistic forms would start first and become dominant, followed by deeper and conceptual processing of word meaning when making a lexical decision. This proposition has found empirical support in an fMRI (functional magnetic resonance imaging) study (Simmons et al., 2008). Similarly, Kelly and Lee (2012) proposed that iconic gestures might be integrated with accompanying speech only *after* the speech system finishes its processing of linguistic forms of words. Therefore, iconic gestures and their accompanying words might not be well integrated in lower level language processing (e.g., recognition of word forms in a lexical decision task) and linguistic forms might be processed prior to the iconic gestures. According to this view, participants in the Gestures-Accompanying-Speech condition would process the presented words prior to recognizing the forms of iconic gestures, resulting in comparable priming effects in both Gestures-Accompanying-Speech and Speech-Alone conditions.

The present study explored the aforementioned possibilities by looking at the priming effects in three conditions: Gestures-Only condition, Speech-Only condition and Gestures-Accompanying-Speech condition, with the types of primes manipulated. Participants were randomly assigned to one of the three conditions and were asked to make lexical decisions to targets that could be words semantically related to iconic gestures, words semantically unrelated to gestures, or non-words. The response times and accuracy rates of the participants in three conditions were examined.

## Method

### Participants

One-hundred and eighty undergraduates (85 males; ages 18–21 years old) from the National University of Singapore participated in this experiment. They all had correct or corrected-to-normal vision and were native English speakers^1^. They were either awarded course credit or a voucher of SGD$5 for participation.

### Materials

#### Primes

Forty gesture primes were used in the Gestures-Only condition. These gestures were the same as those used in Yap et al.'s (2011) study. They were shown in the video clips in which the person who gestured was clearly seen. In their study, Yap et al. asked a separate group of 45 English-speaking undergraduates from the National University of Singapore to watch 80 silent videotaped gestures, each lasting for 3–4 s on a computer screen in a speechless context, and to write a single word that best described the meaning of a gesture. A gesture whose meaning was agreed upon by 70% of the participants was considered to have a common interpretation (see Goh et al., 2009). Using this criterion, 40 gestures (out of 80) had consistency rates of 70% of above and thus were selected to become gesture primes. The present study used all these forty gesture primes, with each gesture clip 1000 ms long (Yap et al., 2011; Experiment 2). Appendix Table A1 contains a list of gestures and their associated meanings based on participants' interpretation in Yap et al.'s (2011) study.

We then used the gesture labels provided by the participants in Yap et al.'s (2011) to create the prime stimuli in the Speech Only condition. Forty auditory clips, which contained vocal tokens conveying the same semantic meaning as the accompanying gestures, were made. For example, a vocal token of “*rabbit*” that matched the gesture video clip of RABBIT (i.e., the index and middle fingers of both hands are flexed and un-flexed above the head) was recorded. The average duration of auditory clips varied across different vocal tokens (*M* = 655.13 ms, *SD* = 81.96 ms). All vocal tokens were recorded by a linguistically trained female speaker. A *post-hoc* test on the intelligibility of the vocal tokens was conducted on a group of 30 participants. The average agreement rate was 95.92%. However, two out of the 40 vocal tokens had agreement rates below 70%^2^ (see Appendix Table A2 for a list of vocal tokens and their intelligibility rates).

The prime stimuli in the Gestures-Accompanying-Speech condition reflected the simultaneous coupling of gesture and audio clips. Both gesture and its corresponding speech started at the same time. Therefore, participants would watch the gesture videos while listening to the auditory inputs. Each gesture + speech clip lasted for 1000 ms.

#### Targets

The lexical targets consisted of words and non-words. Lexical words were derived from the interpretation of gestures given by participants in Yap et al.'s (2011) study. Then they were matched with the words listed in Nelson et al.'s (1998) free association norms. The strongest associate in the Nelson et al. norms for each lexical target was chosen. The non-words were then created by matching their length, number of orthographic neighbors, and number of syllables based on the ARC Non-word Database (Rastle et al., 2002) and the English Lexicon Project (Balota et al., 2007). Each participant was presented with 20 word and 20 non-word targets. Of the word targets, half were semantically related to the primes (gestures, vocal tokens, or both) and half were not.

### Procedure

All stimuli were presented on computers using the E-Prime 2.0 software (Schneider et al., 2002). Participants in all conditions were instructed to classify letter strings as words and non-words, where they pressed “/” on the keyboard for a word and “z” for a non-word.

The participants were given ten practice trials before the actual experiment. Each trial began with a black fixation sign (#) located at the center of the screen that was displayed for 1000 ms followed by a blank white screen for 200 ms. A prime was then presented to the participants. It was a gesture clip shown on a screen (Gestures-Only condition), an audio clip played binaurally through the headphones connected to a computer (Speech-Only condition), or a combination of both (Gestures-Accompanying-Speech condition). The prime type was manipulated between subjects. After the prime, a blank white screen was shown for 200 ms. Finally, a lexical target, in black lowercase letter, was displayed at the center of the screen for 3000 ms or until participants made a lexical decision. A blank white screen was shown for 1000 ms for correct responses, while a signal of “Incorrect answer” was shown for 1000 ms for incorrect answers. A signal of “No response” was shown if participants did not make a decision after 3000 ms.

The experiment ended after participants had completed all forty trials (10 related and 10 unrelated prime-target pairs, and 20 non-words).

The primes in the three conditions (related, unrelated, and non-word) were counterbalanced across participants such that each prime had an equal chance of appearing in each of the three conditions. The order of the trials was also randomized anew for every participant. Accuracy rates and responses latencies of lexical decisions were recorded.

## Results

Accuracy was almost at ceiling in all three conditions. However, four participants who scored below 85% in accuracy were removed from the analyses (one from the Gestures-Only condition, one from the Speech-Only condition, and two from the Gestures-Accompanying-Speech condition). A box-plot analysis was conducted on the mean response latencies of the participants to identify any outlier participants. Errors (4.1% for “Gesture” condition, 3.7% for “Auditory” condition and 2.8% for “Gesture + Auditory” condition) and response latencies faster than 200 ms or slower than 3000 ms were excluded from the analyses. Response latencies more than 2.5 *SD*s above or below each participant in each condition were excluded from the analyses as well (2.4% for the Gestures-Only condition, 2.6% for the Speech-Only condition and 2.4% for the Gestures-Accompanying-Speech condition). This removed a further 2.5% of the responses.

**Figure 1:**
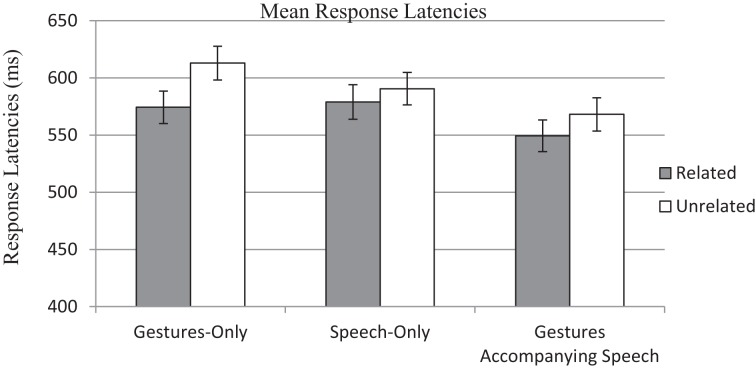
**The mean response latencies to semantically related words (gray bars) and to semantically unrelated words (white bars) in three conditions**.

A 2 (Relatedness: Related vs. Unrelated) × 3 (Conditions: Gestures-Only, Speech-Only and Gesture Accompanying Speech) mixed analysis of variance (ANOVA) was conducted on response latencies and accuracy rates separately. Figure 1 shows the mean response latencies and standard errors in three conditions. Regarding the analyses on response latencies, the main effect of Relatedness was significant by both participants, *F*_*p*(1, 173)_ = 30.08, *p* < 0.001, η^2^_*p*_ = 0.15; and items, *F*_*i*(1, 39)_ = 5.26, *p* = 0.027, η^2^_*p*_ = 0.12. Overall, response latencies were faster when primes and targets were semantically related (*M* = 566.26 ms, *SD* = 160.80 ms) compared to when they were not (*M* = 590.24 ms, *SD* = 161.43 ms). The main effect of Condition was significant for items, *F*_*i*(2, 78)_ = 18.21, *p* < 0.001, η^2^_*p*_ = 0.32, but not significant for participants, *F*_*p*(2, 173)_ = 1.67, *p* = 0.190, η^2^_*p*_ = 0.02. Response latencies in the Gestures-Only condition (*M* = 591.22 ms, *SD* = 174.63 ms), Speech-Only condition (*M* = 585.28, *SD* = 162.06 ms) and Gestures-Accompanying-Speech condition (*M* = 558.26 ms, *SD* = 144.79 ms) were not significantly different from each other.

The interaction between Relatedness and Condition was significant by participants, *F*_*p*(2, 173)_ = 3.73, *p* = 0.026, η^2^_*p*_ = 0.04, but not significant by items, *F*_*i*(2, 78)_ = 1.17, *p* = 0.317, η^2^_*p*_ = 0.03. Bonferonni pairwise comparisons were then conducted to examine whether all three conditions showed significant priming effects. The priming effect in the Gestures-Only condition was significant, *M* = 38.66, *SD* = 54.71, *F*_(1, 173)_ = 28.44, *p* < 0.001, Cohen's *d* = 5.33, same as that in the Gestures-Accompanying-Speech condition, *M* = 18.76, *SD* = 58.28, *F*_(1, 173)_ = 6.59, *p* = 0.006, Cohen's *d* = 2.57. However, the priming effect in the Speech-Only condition was marginally significant, *M* = 11.65, *SD* = 54.02, *F*_(1, 173)_ = 2.58, *p* = 0.055, Cohen's *d* = 1.61.

Following this, we compared the strength of priming effects across different conditions. A *post-hoc* test (LSD) revealed that the priming effect in the Gestures-Accompanying-Speech condition (*M* = 18.76 ms, *SD* = 58.28 ms) was significantly weaker than that in the Gestures-Only condition (*M* = 38.66 ms, *SD* = 54.71 ms), *p* = 0.028, but was comparable to that in the Speech-Only condition (*M* = 11.65 ms, *SD* = 54.02 ms), *p* = 0.245. The priming effect in the Gestures-Only condition was significantly stronger than that in the Speech-Only condition, *p* = 0.005.

### Accuracy

Table 1 shows the mean accuracy rates and standard deviations in three conditions. The main effect of Relatedness was not significant by participants *F*_*p*(1, 173)_ = 0.66, *p* = 0.419, η^2^_*p*_ = 0.00; and items, *F*_*i*(1, 39)_ = 0.84, *p* = 0.366, η^2^_*p*_ = 0.02. The main effect of Condition was also not significant by participants, *F*_*p*(2, 173)_ = 0.79, *p* = 0.456, η^2^_*p*_ = 0.01; and items, *F*_*i*(2, 78)_ = 0.98, *p* = 0.382, η^2^_*p*_ = 0.02. The interaction between Relatedness and Condition was also not significant by participants, *F*_*p*(2, 173)_ = 0.06, *p* = 0.939, η^2^_*p*_ = 0.00; and items, *F*_*i*(2, 78)_ = 0.07, *p* = 0.933, η^2^_*p*_ = 0.00.

**Table 1:** **The mean (and standard deviation) of response accuracies in lexical decision of semantically related and unrelated words in three conditions**.

	**Mean**	***SD***	**Mean**	***SD***
	**Related**		**Unrelated**	
Gestures-only	0.977	0.064	0.974	0.056
Speech-only	0.984	0.039	0.979	0.061
Gestures-accompanying-speech	0.986	0.034	0.984	0.042

## Discussion

Our study set out to examine whether iconic gestures accompanying speech prime semantically-related words and to compare priming in this condition to when gestures or speech are presented alone. The findings revealed that the priming effect in the Gestures-Accompanying-Speech condition was significant but it was weaker than that in the Gestures-Only condition and comparable to that in the Speech-Only condition. We will first discuss the findings in the Gestures-Only and Speech-Only conditions, followed by that those in the Gestures-Accompanying-Speech condition.

The results in the Gestures-Only condition replicated the findings in Yap et al.'s (2011) study, showing that iconic gestures prime semantically related target words in a lexical decision task. These results are consistent with previous research that support the semantic link between the gestural and lexical representational systems. The forms of iconic gestures tested in the present study (also in Yap et al., 2011) might possess a relatively direct and transparent relationship with their meaning such that they were well recognized despite the fact they were presented without speech. Hence, the rich imagistic meaning of iconic gestures would provide useful semantic information and allow participants to activate semantically related words. A significant priming effect was also found in the Speech-Only condition. This finding converges with previous research (Holcomb and Neville, 1990; Holcomb and Anderson, 1993), and demonstrates that cross-modal priming from an auditory prime to a visual target is reliably obtained across different studies. The foregoing results demonstrate that cross-modal priming can be generalized across different modalities (i.e., gesture-visual as well as auditory-visual). This reinforces the idea that our semantic representation is represented in multiple formats, such as gestures, audio tokens, and words (Krauss, 1998).

Although it was not one of our major predictions, we found that the priming yielded by iconic gestures presented alone was stronger than that of speech alone and that of speech accompanied by iconic gestures. What might account for this? It is possible that processing iconic gestures presented alone might result in a deeper semantic processing than processing words and words accompanied by iconic gestures. Since iconic gestures do not have conventional labels and standardized forms (McNeill, 2000), they might be connected to a wider range of semantic concepts than words, thus strengthening priming. In contrast, words have conventional labels and can consolidate the meanings of accompanying iconic gestures. We acknowledge that the foregoing proposal is *post-hoc* and speculative, given that no study to date has directly compared the degree of semantic processing engaged by iconic gestures (either presented alone or co-occurring with speech) and words in a lexical decision task. This will be an interesting research direction to pursue in the future.

However, one might contend that iconic gestures presented alone are less constrained in their meanings, and thus participants would have to work harder in order to process the meanings of gesture, compared to spoken word, primes. Previous work have established that larger priming effects are produced when more time is taken to process a prime (Hutchison et al., 2008). That said, we should emphasize that the gesture primes we have selected for the present study (and also Yap et al.'s study in 2011) received a relatively high agreement rate (70%) in a norming study. Note that this is the same threshold adopted when one decides whether a particular auditory token can be correctly identified by a subject. While we cannot entirely exclude the possibility that the extra effort involved in processing gestures might induce stronger priming, we have attempted to minimize this possibility by choosing only high-agreement gesture primes.

Our findings also showed that gestures accompanying speech prime semantically related words. While previous studies have focused primarily on the priming effects when iconic gestures were presented alone (Bernardis et al., 2008; Yap et al., 2011), our results extend the literature by demonstrating that such priming also occurs even in a naturalistic setting where iconic gestures are not presented alone, but co-occurring with speech.

Intriguingly, the priming effect in the Gestures-Accompanying-Speech condition was comparable in size to that in the Speech-Only condition. However, this finding can be well explained by the Theory of Language and Situated Simulation (LASS; Barsalou et al., 2008). According to this theory, making lexical decisions (i.e., discriminating words from non-words) can be mainly driven by the processing of linguistic forms alone, without accessing deep conceptual meaning. In addition, the linguistic system dominates the early stage of conceptual processing (Simmons et al., 2008). Because of the *asynchrony* in the time-course of linguistic (first) and conceptual (second) processing, the facilitating effect of co-speech iconic gestures might therefore be weakened. Participants in the Gestures-Accompanying-Speech condition (as well as the Speech-Only condition) might mainly drive their lexical decision from activity in the linguistic system. However, we want to be careful to point out that we are *not* arguing that participants did not process the deep conceptual information of the presented targets. Instead, the speech and iconic gestures presented in the Gestures-Accompanying-Speech would actually activate associated simulations (Barsalou et al., 2008). However, such activation might occur *after* the linguistic system has recognized the presented words, at which point lexical decisions have already been made.

The comparable priming effects in the Gestures-Accompanying-Speech and Speech-Alone conditions might seem to be inconsistent with the extant literature, which demonstrates the facilitatory effect of gestures on processing when they accompany speech (e.g., Beattie and Shovelton, 1999; Cohen and Otterbein, 1992; Kelly et al., 2004; Feyereisen, 2006; Özyürek et al., 2007). However, the tasks in which participants engaged in previous studies were not lexical decision tasks; rather they involved language comprehension and memory. Our findings thus shed light on the role of co-speech iconic gestures in facilitating word recognition. Specifically, the facilitatory effect of co-speech iconic gestures is more salient in higher level language processing (e.g., understanding conceptual meaning of words) than in lower level of language processing (e.g., discriminating words from non-words in a lexical decision task).

One might also contend that the comparable priming effect in the Gestures-Accompanying-Speech and Speech-Alone conditions was attributed to a task-specific effect. In this study, iconic gestures always conveyed the same information as the accompanying speech. Therefore, participants might have realized that iconic gestures did not help them to do better on the task or they even might have stopped attending to the gestures. This could explain why the priming effect in the Gestures-Accompanying-Speech was similar to that Speech-Alone condition. In addition, the non-naturalistic nature of the gestures accompanying speech stimuli (the person who gestured in the video did not say the words) might offer a possible alternative explanation for why the priming effect in the Gestures-Accompanying-Speech condition was less significant than that in the gesture only condition (e.g., subjects may just decide to listen to the speech and ignore the visually presented gesture). However, there is evidence against the idea that participants ignore iconic gestures that reinforce semantic contents of accompanying speech. Specifically, in a study by So et al. (2012), participants watched three different videos, each consisting of a list of 10 words, in 3 conditions (words accompanied by iconic gestures, words accompanied by speech beat gestures, and words not accompanied by gestures). The semantic meaning conveyed in these iconic gestures was the same as that in the accompanying speech. The findings showed that participants recalled more words when encoding them with iconic gestures than when encoding them with speech beats or without gestures. Therefore, presenting iconic gestures that convey the same semantic meaning with accompanying speech might not necessarily prevent participants from attending to these gestures.

In fact, our result is consistent with Kelly and Lee's study (2012) in which they did not find facilitatory effects of co-speech iconic gestures on learning the phonetic forms of a second language. In future work, we could explore the effects of co-speech gestures on a lexical processing task which weights semantic information more heavily, such as the semantic classification task.

To conclude, our study provides additional empirical support for cross-modal semantic priming. That is, gestures presented alone or with speech prime semantically related words in a lexical decision task. However, the priming effect of iconic gestures accompanying speech is weaker than that of iconic gestures presented alone, suggesting that the facilitatory effect of iconic gestures may be limited in tasks which rely more heavily on linguistic, compared to semantic, processing.

### Conflict of interest statement

The authors declare that the research was conducted in the absence of any commercial or financial relationships that could be construed as a potential conflict of interest.

## Footnotes

^1^In Singapore, English is the medium of instruction at all levels of education and is also the major official language. The last author of this manuscript routinely collect standardized vocabulary knowledge scores from his participant (Shipley, 1940). Participants in the National University of Singapore have a mean Shipley vocabulary age of about 18 (Yap et al., 2013), which is lower than the mean vocabulary age of a highly selective North American college (Washington University: 18.75) but higher than that of a typical state college (SUNY-Albany: 17.25) (Yap et al., 2009).

^2^The removal of these two vocal tokens from our analyses did not result in any significant differences in our results or discussion.

## Appendix

**Table A1:** **List of gestures and their associated meanings as given by participants**.

**Gesture meaning (Yap et al., 2011)**	**Lexical targets (Nelson et al., 1998)**	**Prime-target associative strength (Nelson et al., 1998)**	**Non-words (ARC; ELP)**
Baby	Child	0.20	Youns
Ball	Round	0.15	Sorms
Brush	Hair	0.20	Salm
Cap	Hat	0.06	Cug
Carry	Hold	0.02	Lown
Circle	Square^*^	0.47	Blurds
Climb	Mountain	0.09	Sprounge
Cold	Hot	0.67	Tib
Comb	Brush	0.16	Vonks
Curve	Straight	0.08	Phlieves
Cut	Blood	0.02	Skent
Cycle	Bike	–^**^	Mout
Down	Up	0.84	Vu
Drive	Car	0.12	Dar
Fat	Skinny	0.41	Aggous
Fill	Empty^*^	–^**^	Chuzz
Fly	Bird	0.32	Vuch
Full	Empty^*^	0.61	Slolt
Grab	Take	0.03	Jole
Guitar	String	0.06	Terked
Hot	Cold	0.41	Boke
Open	Close	0.44	Nubes
Photo	Picture	0.06	Iddings
Pray	God	–^**^	Gos
Push	Pull	0.59	Pome
Rabbit	Bunny	0.73	Trief
Read	Book	0.42	Teap
Rectangle	Square^*^	–^**^	Blurds
Run	Walk	0.46	Joth
Slap	Hit	0.02	Cug
Stir	Mix	0.19	Zix
Swim	Water	0.05	Aiped
Think	Brain	0.23	Polfs
Throw	Ball	0.07	Sogs
Tie	Neck	0.07	Tesh
Tiny	Small	0.08	Crosh
Triangle	Square^*^	0.04	Quescs
Up	Down	0.58	Datt
Walk	Run	0.49	Ven
Write	Read	0.33	Dunt

* The repeated stimuli were not presented in the same block within participants after counterbalancing.

** No values were reported for these stimuli because they were selected to fit the additional cues or information conveyed by the gestures and were therefore not found in Nelson et al.'s (1998) norms.

**Table A2:** **List of vocal tokens and their participant agreement rate**.

**Vocal tokens**	**Agreement rate (%)**
Baby	100
Ball	93.33
Brush	100
Cap	93.33
Carry	100
Circle	100
Climb	96.67
Cold	100
Comb	100
Curve	100
Cut	100
Cycle	100
Down	100
Drive	96.67
Fat	86.67
Fill	83.33
Fly	100
Full	53.33^*^
Grab	96.67
Guitar	100
Hot	100
Open	100
Photo	100
Pray	93.33
Push	100
Rabbit	100
Read	96.67
Rectangle	100
Run	100
Slap	96.67
Stir	93.33
Swim	100
Think	96.67
Throw	100
Tie	96.67
Tiny	100
Triangle	100
Up	100
Walk	100
Write	63.33^*^

* There were two vocal tokens with an agreement rate below 70%.
